# Gastric emptying is rapid in adolescents with type 1 diabetes and relates to gastrointestinal symptoms

**DOI:** 10.1186/1687-9856-2015-S1-O46

**Published:** 2015-04-28

**Authors:** Shiree Perano, Christopher Rayner, Stamatiki Kritas, Christine Mpundu-Kaambwa, Kim Donaghue, Michael Horowitz, Jennifer Couper

**Affiliations:** 1 Diabetes and Endocrinology Department, Women’s and Children’s Hospital, North Adelaide, SA, Australia; 2 University of Adelaide, Adelaide, SA, Australia; 3 Gastroenterology Department, Royal Adelaide Hospital, SA, Australia; 4 Gastroenterology Department, Women’s and Children’s Hospital, North Adelaide, SA, Australia; 5 Research and Evaluation Unit, Women’s and Children’s Hospital, North Adelaide, SA, Australia; 6 Institute of Endocrinology, Children’s Hospital Westmead, Sydney, NSW, Australia; 7 Endocrinology Department, Royal Adelaide Hospital, North Terrace, SA, Australia

## 

Gastric emptying is a critical determinant in postprandial glycaemic control. This study aimed to assess whether gastric emptying in adolescents with type 1 diabetes (T1D) relates to gastrointestinal symptoms and to heart rate variability (HRV) as a measure of autonomic function.

We studied 30 adolescents (age 15 ± 2.5 years, BMI 22 ± 3.1 kg/m^2^) with T1D. Subjects consumed a ^13^C labelled pancake meal. Gastric emptying was measured by ^13^C breath test. Blood glucose was monitored frequently over 4 hours and gastrointestinal symptoms at 30-60 minute intervals, by a visual analogue questionnaire. Chronic gastrointestinal symptoms over the previous 3 months were assessed by a validated Diabetes Bowel Symptoms Questionnaire [1]. HRV was assessed by LabChart Pro [2].15 age and sex matched controls were also studied.

Gastric half emptying time was accelerated in adolescents with T1D compared to controls; 77.6 (61.4-99.3) minutes [median (IQR)] versus 109.1 (70.8-124.2), P = 0.02), independent of hyperglycaemia during the study, HbA1c, duration of diabetes, and BMI.

There was no difference in the prevalence of chronic symptoms or symptoms of a severity that affected lifestyle between the two groups.

The presence of nausea, vomiting, bloating and/or fullness during the study in T1D was associated with faster gastric emptying compared to asymptomatic T1D (r =0.55; p = 0.04), and this was independent of peak glucose and glucose at 4 hours.

Rate of gastric emptying in T1D did not correlate with HRV.

Adolescents with T1D have rapid gastric emptying associated with acute gastrointestinal symptoms. Symptomatology could be used as a clinical tool to determine the need for further investigation.

## References

[B1] Quan C Talley NJ Cross S Jones M Hammer J Giles N Development and validation of the Diabetes Bowel Symptom Questionnaire. Aliment Pharmacol Ther 2003 17 9 1179 1187 10.1046/j.1365-2036.2003.01553.x 12752355

[B2] Task Force of the European Society of Cardiology and the North American Society of Pacing and Electrophysiology. Heart rate variability: standards of measurement, physiological interpretation and clinical use. Circulation 1996 93 5 1043 1065 10.1161/01.CIR.93.5.1043 8598068

